# Recent advances in precision medicine for pancreatic ductal adenocarcinoma

**DOI:** 10.1002/ags3.12436

**Published:** 2021-02-03

**Authors:** Hiromitsu Hayashi, Takaaki Higashi, Tatsunori Miyata, Yo‐ichi Yamashita, Hideo Baba

**Affiliations:** ^1^ Department of Gastroenterological Surgery Graduate School of Life Sciences Kumamoto University Kumamoto Japan

**Keywords:** chemotherapy, genetic mutation, molecular subtype, pancreatic ductal adenocarcinoma, precision medicine

## Abstract

Pancreatic ductal adenocarcinoma (PDAC) is one of the leading causes of cancer mortality worldwide. Although advances in systemic chemotherapy for PDAC have improved survival outcomes for patients with the disease, chemoresistance is a major treatment issue for unselected PDAC patient populations. The existence of heterogeneity caused by a mixture of tumor cells and stromal cells produces chemoresistance and limits the targeted therapy of PDAC. Advances in precision medicine for PDACs according to the genetics and molecular biology of this disease may represent the next alternative approach to overcome the heterogeneity of different patients and improve survival outcomes for this poor prognostic disease. The genetic alteration of PDAC is characterized by four genes that are frequently mutated (*KRAS*, *TP53*, *CDKN2A*, and *SMAD4*). Furthermore, several genetic and molecular profiling studies have revealed that up to 25% of PDACs harbor actionable alterations. In particular, DNA repair dysfunction, including cases with *BRCA* mutations, is a causal element of sensitivity to platinum‐based anti‐cancer agents and poly‐ADP ribose polymerase (PARP) inhibitors. A deep understanding of the molecular and cellular crosstalk in the tumor microenvironment helps to establish scientifically rational treatment strategies for cancers that show specific molecular profiles. Here, we review recent advances in genetic analysis of PDACs and describe future perspectives in precision medicine according to molecular subtypes or actionable gene mutations for patients with PDAC. We believe the breakthroughs will soon emerge to fight this deadly disease.

## Graphical abstract

Advances in precision medicine for PDACs according to the genetics and molecular biology of this disease may represent the next alternative approach to overcome the heterogeneity of different patients and improve survival outcomes for this poor prognostic disease. Several genetic and molecular profiling studies have demonstrated that up to 25% of PDACs harbor actionable molecular alterations. Here, we review recent advances in genetic analysis of PDACs and describe future perspectives in precision medicine according to molecular subtypes or actionable gene mutations for patients with PDAC.

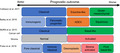

## 1 INTRODUCTION

Pancreatic ductal adenocarcinoma (PDAC) remains the most lethal type of cancer.^1^ Both GnP (gemcitabine plus nab‐paclitaxel) and FOLFIRINOX (5‐fluorouracil, folinic acid, irinotecan, and oxaliplatin) regimens have improved survival outcomes of patients with metastatic PDAC.^2^, ^3^ For resected PDAC, median overall survival (OS) has also increased from 22.1 to 35 months during the past 10 years, largely due to improvements in adjuvant therapies.^4^, ^5^, ^6^, ^7^, ^8^, ^9^ On the other hand, the high recurrence rate even in patients who have undergone curative resection and chemoresistance for the current systemic chemotherapies (GnP and FOLFIRINOX) are major issues in the treatment of unselected PDAC patient populations. Although molecular markers are often employed to effectively select patients for anti‐cancer agents, only imaging modalities are applied to stage the disease and judge suitability for operative resection. Unfortunately, our knowledge of the genetic and biological backgrounds of this deadly disease has not yet been linked to a leap in patient survival. Knowledge obtained from the Human Genome Project, and subsequently The Cancer Genome Atlas, has yielded the landscape of precision medicine. The concept is that cancer patients can be sub‐classified according to actionable driver mutations, which can be targeted by molecular‐specific agents. Development of next‐generation sequencing (NGS) has drastically progressed genomic sequencing technology and cleared actionable driver mutations for individual cancer patients. Advances in precision medicine for PDACs according to genetics and molecular biology may be the next alternative approach to improve survival outcomes for this poor prognostic disease. PDACs have been divided into several molecular subtypes by recent advances in genetic analysis,^10^, ^11^, ^12^, ^13^, ^14^, ^15^, ^16^, ^17^ which is a precursor of precision medicine. Some molecular profiling studies have exhibited that up to 25% (range 12%‐25%) of PDACs retained actionable molecular alterations.^10^, ^11^, ^12^, ^13^, ^14^, ^15^, ^16^, ^17^ Furthermore, the development of multigene panel assay has resulted in a fundamental change in the treatment of PDAC. Indeed, matching to appropriate molecular‐specific treatments improves the OS of PDAC patients compared to that of those without actionable mutations or those who do not accept the molecular‐specific therapy.^18^ A better grasp of the genetics and molecular biology of PDAC accelerates the development of precision medicine.

Here, we review recent advances in genetic analysis of PDACs and describe future perspectives in precision medicine according to molecular subtypes or gene mutations for patients with PDAC.

## 2 THE GENOMIC LANDSCAPE OF PANCREATIC CANCER AND “BIG FOUR” MUTATION GENES

In 2008, the exome analysis of PDACs was completed.^19^ The coding regions of >20 000 genes were sequenced, and an average of 63 genomic alterations per patient genome was discovered. These alterations consisted of 12 core signaling pathways and were detectable in the majority (from 67% to 100%) of PDACs. Among them, dysregulations in KRAS signaling, G1/S phase cell cycle transition, TGF‐β signaling, integrin signaling, cell invasion, homophilic cell interaction, and small guanine triphosphate (GTPase)‐dependent signaling were prominent.^19^

**FIGURE 1:**
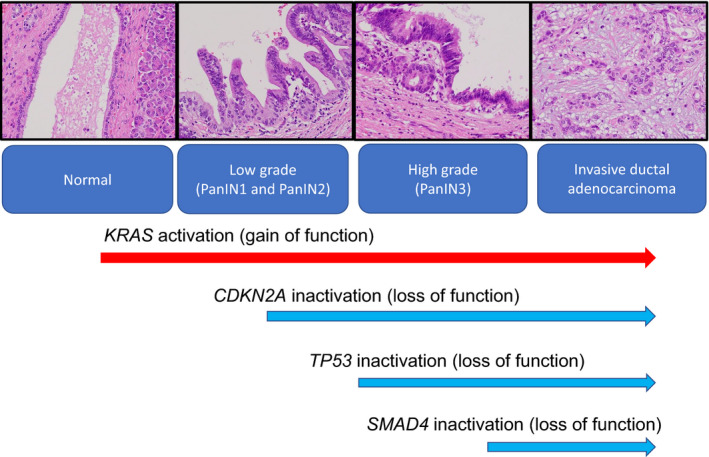
Progression of pancreatic ductal adenocarcinoma development from pancreatic intraepithelial lesions (PanINs) and genetic alterations. The lower‐grade lesions (PanIN1 and PanIN2) frequently retain genetic alterations in *KRAS* and *CDKN2A* genes, while the higher‐grade lesions (PanIN3) exhibit the additional mutations in *TP53* and *SMAD4* genes. Progression of PanINs correlates with sequentially accumulating genetic mutations

The genetic landscape of PDACs is featured by four frequently mutated genes: *KRAS*, *TP53*, *CDKN2A* (p16), and *SMAD4*.^20^ The four predominant gene mutations appear to occur sequentially as PanIN progresses (Figure 1). *KRAS* mutations can be found even in normal pancreases and in PanIN1. In PDAC, the incidence of oncogenic *KRAS* mutation ranges from 88% to 100%.^12^, ^16^, ^17^, ^19^, ^21^, ^22^ Although the initial step for PDAC development remains to be elucidated, the oncogenic *KRAS* mutation is a key event, as evidenced by its presence in PanIN lesions^23^, ^24^ and the development of PanIN lesions in oncogenic *KRAS*‐driven GEMMs.^25^, ^26^ The oncogenic *KRAS* mutation provokes the constitutively activated RAS protein and results in the dysregulated activation of proliferation and survival pathways. In the clinical setting, cases with *KRAS* mutations displayed worse prognostic outcome with a median survival time of 17 months compared to 30 months for those without mutations.^27^ In analysis of *KRAS* mutation type, codon G12D mutant was the most frequent (48%), followed by G12V (31%) and G12R (21%).^22^ Intriguingly, 4% of PDACs exhibit multiple *KRAS* mutations, and these different *KRAS* mutations appeared in distinct cancer cells in a single tumor.^22^ While G12D or G12V mutations are the most prevalent *KRAS* mutations in patients with PDAC, codon G13 and Q61 mutations have also been noted.^12^, ^17^, ^19^, ^28^ The point mutations in codon 12, 13, or 16 result in reduced GTP hydrolysis. In contrast, cases with *KRAS* mutations at codon 61 revealed a favorable prognosis, as they display weaker ERK activation.^17^ Thus, different *KRAS* mutations induce diverse signaling activities with distinct biological impacts.^29^, ^30^ Small‐molecule inhibitors targeting KRAS^G12C^, a mutation exhibited in ~1.5% of PDAC cases, display encouraging anti‐cancer effects against solid tumors including PDAC in vitro and in vivo.^31^ *CDKN2A* is inactivated by mutation of alleles, homozygous deletion, or promoter hypermethylation in 75%–95% of PDAC cases.^20^, ^32^ Inactivation of the *CDKN2A* gene induces the loss of p16 protein, which is a negative regulator of the G1/S transition of the cell cycle, and then promotes proliferative activity. *TP53* encodes the p53 tumor suppressor protein, which is responsible for retaining genetic and cellular stability. TP53 protein regulates the cell cycle at the G1/S interface and plays a crucial role in provoking programmed cell death in reaction to DNA damage. *TP53* is the most frequently mutated gene in cancer.^33^ *TP53* inactivation allows the cell with DNA damage to evade important checkpoints to trigger apoptosis. It is mutated (mainly by point mutations) in 75%–85% of PDAC cases.^20^, ^34^ *SMAD4* encodes Smad4 protein, which is a transcription factor in TGF‐β signaling pathway.^35^ SMAD4 is inactivated in 43%–50% of PDAC cases.^11^, ^20^ It works with TGF‐β1 as a tumor suppressor to regulate cell cycle arrest and apoptosis.^36^ The loss of *SMAD4* gene induces aberrant TGF‐β signaling. PDAC patients with biallelic deletion of *SMAD4* had more frequent metastasis compared to those with wild‐type *SMAD4*.^37^ Lower‐grade lesions (PanIN1 and PanIN2) frequently retain genetic alterations in *KRAS* and *CDKN2A* genes, while the higher‐grade lesions (PanIN3) exhibit the additional mutations in *TP53* and *SMAD4* genes.

Yachida et al^38^ showed data that the number of mutations among the major four driver genes was substantially associated with OS and disease‐free survival (DFS). Among 79 PDAC patients, one (1%) had a single gene alteration, 14 (18%) had two gene alterations, 35 (44%) had three gene alterations, and 29 (37%) had an alteration in all four genes. The increased number of altered genes was significantly associated with worse DFS and OS at autopsy.^38^ Additionally, Hayashi et al^39^ reported that PDAC patients with fewer mutations displayed a better prognostic outcome in 71 patients who underwent a radical operation followed by adjuvant chemotherapy. The existence of zero to two mutated genes was a predictor of a better OS.^39^ Furthermore, genetic alterations of three genes (except *KRAS*), and thereby protein overexpression in PDAC tissues, are associated with malignant activity of PDAC.^40^ In particular, loss of SMAD4 immunolabeling was an independent poor prognostic factor for OS and DFS in patients with resectable PDAC.^40^ Intriguingly, all of the six patients who achieving 5‐year survival displayed intact SMAD4 expression. Thus, the genetic status of the so‐called “big four” mutation genes or their immunolabeling status is a prognostic biomarker in PDAC patients. Unfortunately, there is still no available drug that can directly target the major four gene mutations in PDACs.

## 3 MOLECULAR SUBTYPE CLASSIFICATION

**FIGURE 2:**
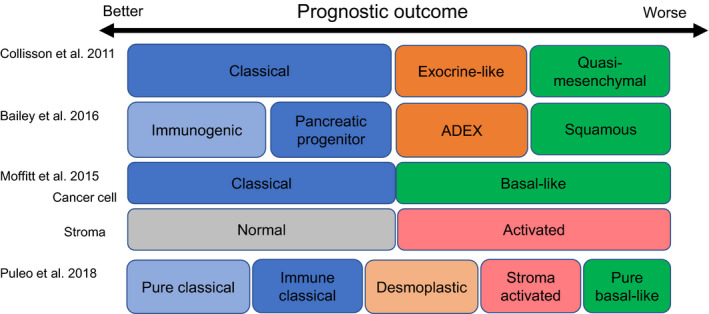
Molecular classification and prognostic relevance in pancreatic ductal adenocarcinoma. ADEX, aberrantly differentiated endocrine exocrine

Recent advances in biotechnology enable us to execute comprehensive genomic, transcriptomic, proteomic, and metabolomic analyses rapidly and cheaply. Such comprehensive gene expression studies have recognized subtypes of PDAC with biological and prognostic relevance (Figure 2, Table 1).

**TABLE 1:** Subtype classification of pancreatic ductal adenocarcinomas and their prognostic impacts

Classification	MST (M, months)	Molecular or clinical features
**Collisson et al** ^13^		
*Tumor classification into three subtypes*		
Classical (n = 14, 52%)	Better (786 d in mean)	GATA6↑, sensitive to erlotinib (in vitro)
Exocrine‐like (n = 5, 18%)	Moderate (564 d in mean)	
Quasi‐mesenchymal (n = 8, 30%)	Worse (304 din mean)	Sensitive to gemcitabine (in vitro)
**Moffitt et al** ^15^		
*Tumor‐specific subtypes into two types*		
Classical (n = 89)	19M (70% 1 y survival)	GATA6↑
Basal‐like (n = 36)	11M (44% 1 y survival)	Better response to adjuvant therapy
*Stroma‐specific subtypes into two types*		
Normal (n = 30)	24M (82% 1 y survival)	ACTA2↑, VIM↑, DES↑ (stellate cells)
Activated (n = 78)	15M (60% 1 y survival)	ITGAM↑, CCL13↑, CCL18↑ (macrophages)
Classical and normal	0.39 (lowest hazard ratio of death) (0.21–0.73 in 95%CI)	
Basal and activated	2.28 (highest hazard ratio of death) (1.34–3.87 in 95%CI)	
**Bailey et al** ^11^		
*Tumor classification into four subtypes*		
Immunogenic (classical)	30.0M	immune suppression
Pancreatic progenitor (classical)	25.6M	pancreatic development (FOXA 2/3↑, PDX1↑, MNX1↑)
ADEX (exocrine like)	23.7M	KRAS activation, exocrine (NR5A2↑ and RBPJL↑) endocrine differentiation (NEUROD1↑ and NKX2‐2↑)
Squamous (QM or Basal)	13.3M	*TP53* mutation, *KDM6A* mutation, TP63ΔN transcriptional network↑, hypermethylation of pancreatic endodermal cell fate‐determining genes (for example, PDX1, MNX1, GATA6, HNF1B)
**Puleo et al** ^41^		
*Tumor classification into five subtypes*		
Pure classical (n = 70)	43.1M	Low stromal signal, well differentiated tumor
		*KRAS* mutation (G12R), high hENT1 expression
Immune classical (n = 25)	37.4M	Significant stromal signature, structural vascularized and immune stroma,
		High hENT1 expression
Desmoplastic (n = 67)	24.3M	Low cell component and a marked stromal transcriptomic signal
Stroma activated (n = 54)	20.2M	Activated stromal component explained by high a‐SMA, SPARC, and FAP,
		*CDKN2A* mutation, *TP53* mutation
Pure basal‐like (n = 25)	10.3M	Low stromal signal, poorly differentiated tumor,
		*KRAS* mutation (G12D), *KRAS* (G12V), nuclear GLI1 expression,
		*CDKN2A* mutation, *TP53* mutation

Validation of subtype classification for PDACs		
**Aung et al** ^20^		
*According to Moffitt's “tumor” subtypes*		
Classical (76%)	10.4M	GATA6↑, HNF4A ↑, sensitive to cisplatin
		Partial response to chemotherapy in 34%
Basal‐like (24%)	6.3M	Hypoxia↑, metastatic pathways↑ partial response to chemotherapy in 8%
Classical subtype treated with modified FOLFIRINOX		Best PFS 8.5M (6.5M not reached in 95%CI)
Basal‐like subtype treated with modified FOLFIRINOX		Worst PFS 2.7M (2.1M not reached in 95% CI)
**Brinbaum et al** ^42^	**2‐y overall survival**	
*According to Collisson's “tumor” subtypes*		
Classical (n = 263)	45%	
Exocrine‐like (n = 216)	44%	
Quasi‐mesenchymal (n = 122)	25%	
According to Moffitt's “tumor” subtypes		
Classical (n = 365)	48%	
Basal‐like (n = 236)	28%	
*According to Moffitt's “stroma” subtypes*		
Normal (n = 241)	49%	
Activated (n = 333)	34%	
*According to Bailey's subtypes*		
Immunogenic (n = 104)	56%	
Pancreatic progenitor (n = 142)	48%	
ADEX (n = 140)	46%	
Squamous (n = 215)	23%	

Note

Multivariate analysis by including the four classifiers together; Moffitt “stroma” and Bailey classifications show independent prognostic value.

Abbreviations: ADEX, aberrantly differentiated endocrine exocrine; MST, median survival time; PFS, progression‐free survival.

Collisson et al^13^ introduced classification of PDACs into three subtypes: classical, quasi‐mesenchymal (QM), and exocrine‐like. The survival outcome of PDAC cases following surgical resection and standard medical treatment was remarkably better in the classical subtype than that in cases with the QM subtype; cases with the exocrine‐like subtype showed an intermediate survival outcome between the two other subtypes.^13^ Searching the clinical relevance of this classification using PDAC cell lines, the classical and QM subtypes offered differential reactions to gemcitabine and erlotinib.

Moffitt et al^15^ evolved molecular profiling from primary tumors to metastatic and normal lesions. They categorized PDAC tumors into two subtypes (classical and basal‐like). PDAC cases with the basal‐like subtype displayed a worse survival outcome (one‐year survival rate of 44%) compared with 70% for PDAC patients with classical subtype.^15^ Tumor stroma was also categorized into normal and activated. PDAC cases with the activated stromal subtype showed a worse prognosis (a median survival time of 15 months and a one‐year survival rate of 60%) compared to cases with the normal stromal subtype (a median survival time of 24 months and a one‐year survival rate of 82%).^15^ PDAC patients with the classical and the normal stromal subtypes displayed a favorable prognostic outcome, while those with basal‐like and the activated stromal subtypes revealed a very poor prognostic outcome.^15^

Bailey et al executed comprehensive genomic analysis of 456 PDAC cases and determined 32 frequently mutated genes that assemble into 10 pathways such as KRAS, TGF‐β, G1/S transition, DNA repair, WNT, NOTCH, SWISNF, ROBO/SLIT signaling, chromatin modification, and RNA processing.^11^ They classified PDAC tumors into four subtypes in accordance with these gene mutations: squamous, pancreatic progenitor, immunogenic, and aberrantly differentiated endocrine exocrine (ADEX). The squamous subtype is closely associated with *TP53* and *KDM6A* mutations; high TP63 expression is a typical feature of the squamous subtype.^11^ The progenitor subtype is specifically characterized by transcriptional networks including transcription factors FOXA2, FOXA3, HES1, HNF1A, HNF1B, HNF4A, HNF4G, MNX1, and PDX1; these tumors are also featured by TGFBR2‐inactivating mutation and by apomucin expressions such as MUC1, MUC2, and MUC6.^11^ The immunogenic subtype shares several features with the progenitor subtype yet differs in being characterized by the significant infiltration of immune cells.^11^ The ADEX subtype is featured by a transcriptional network such as genes involved in endocrine differentiation and transcription factors involved in acinar cell differentiation.^11^ Except for the immunogenic subtype, three of the four subtypes display similar features with the Collisson classification: thus, the progenitor subtype corresponds to the classical subtype, the ADEX to the exocrine‐like, and the squamous to the QM subtype.^13^

Puleo et al^41^ validated the reported subtypes of PDAC using formalin‐fixed and paraffin‐embedded samples, and then classified them into five subtypes (pure classical, immune classical, desmoplastic, stroma activated, and pure basal‐like) according to the features in both the cancer cells and the tumor microenvironment. The pure basal‐like subtype revealed the worst prognostic outcome with a median OS of 10.3 months, whereas the pure classical showed a good prognostic outcome with a median OS of 43.1 months. Furthermore, they suggested that the previously reported exocrine‐like (ADEX) subtype was due to the contamination with pancreatic acinar cells.

Aung et al^20^ investigated the clinical relevance of Moffitt's tumor subtypes using whole‐genome sequencing (WGS) and RNA sequencing in response to first‐line chemotherapy in advanced PDACs (COSMOSS trial). They reported that the patients with the classical subtype displayed significantly better objective responses to first‐line chemotherapy than those with the basal‐like subtype, and those with the classical subtype treated with modified FOLFIRINOX exhibited the best progression‐free survival (PFS). They concluded that the response to chemotherapy differed among patients with individual subtypes. Brinbaum et al^42^ validated and compared the biological and clinical relevance of the above molecular classifications of PDAC according to the Collisson, Moffitt (tumor and stroma), and Bailey classifications. They investigated the prognostic significance of the Moffitt (stroma) classification and the Bailey classification using multivariate analysis by including the four classifiers together, emphasizing the complementarity of classifiers based on cancer cells and stroma. Rashid et al^43^ developed a classifier for PDAC subtyping to predict subtype in individual patients, based on the three largest bulk gene expression datasets (TCGA PAAD, Aguirre Biopsies, and Moffitt GSE71729). They showed that basal‐like subtype tumors are refractory to FOLFIRINOX‐based regimens.^43^

These innovative systems of reclassifying tumors may guide appropriate treatment decisions.

### 3.1 DNA damage repair pathways

DNA damage is a frequent event and must be immediately repaired to ensure the precise transfer of genetic information during cellular division. Malfunction of the DNA damage repair (DDR) pathway can lead to an accumulation of genomic defects and further impairment of the DNA repair capacity. DNA damage may result from base modifications, single‐strand DNA breaks (SSBs), double‐strand DNA breaks (DSBs), or intrastrand and interstrand DNA crosslinks. Of these, DSBs are the most cytotoxic damages. DNA is repaired by distinct pathways to retain genetic stability. Two principal pathways in DSB repair are homologous recombination (HR), which employs the sister chromatid as a repair template, and non‐homologous end joining (NHEJ), in which a DNA segment is eliminated and both ends are adjoined without attention of homology.^44^ While NHEJ is a more error‐prone form of DSB repair, HR is an accurate process. The MRN (Mre11, Rad50, and Nbs1 proteins) complex acts as the core during the initial DSB repair as an upstream element of HR and partially of NHEJ. The MRN complex fascinates BRCA1 to the DNA damage spot, forming the adjoining 3′ ends and recruiting PALB2 and BRCA2. This complex of BRCA1, BRCA2, and PALB2 activates RAD51, which plays a crucial role for binding single‐stranded DNA segments and mediates to invade into the homologous DNA sequences in the sister chromatid. Thus, BRCA1/2 and PALB2 play a critical role in efficient HR. BRCA1/2‐deficient cells without HR ability store DBSs, which induces genomic instability and increases predisposition to play malignant behaviors.^45^ Although the risk of PDAC in carriers with a mutated *BRCA1* gene is not fully elucidated, it is anticipated to be increased two to threefold over the general population.^46^, ^47^ In contrast, *BRCA2* gene mutation was found in approximately 6% of the same cohort.^48^ The risk of PDAC in carriers with a mutated *BRCA2* gene is reported to be increased three to sixfold over the general population.^49^, ^50^

It is presently anticipated that 17%‐25% of PDACs entertain germline or somatic DDR gene mutations such as *BRCA1/2*, *PALB2*, and ataxia telangiectasia mutated (*ATM*).^10^, ^11^, ^13^, ^14^, ^15^, ^16^, ^17^ Dreyer et al estimated that 24% of PDACs exhibit a DDR deficiency due in 7% of patients to germline mutations of either BRCA1 or BRCA2 or PALB2, in 7% to somatic mutations of these genes, and in the remaining patients to rare mutations of genes such as *ATM*.^51^ Thus, approximately 10%‐20% of PDACs reveal DDR deficiency without *BRCA1/2* mutations (BRCAness). DSBs detected by the MRN complex provoke the serine/threonine kinase ATM. ATM plays a pivotal role in sensing DSBs and triggering machinery to stop the cell cycle until the DNA damage is fixed. Several studies displayed up to 18% of *ATM* mutations in PDACs.^12^, ^16^, ^19^, ^23^, ^52^ In a mouse model of PDAC, ATM deficiency accelerates genomic instability and metastatic ability.^53^

### 3.2 Platinum sensitivity

**FIGURE 3:**
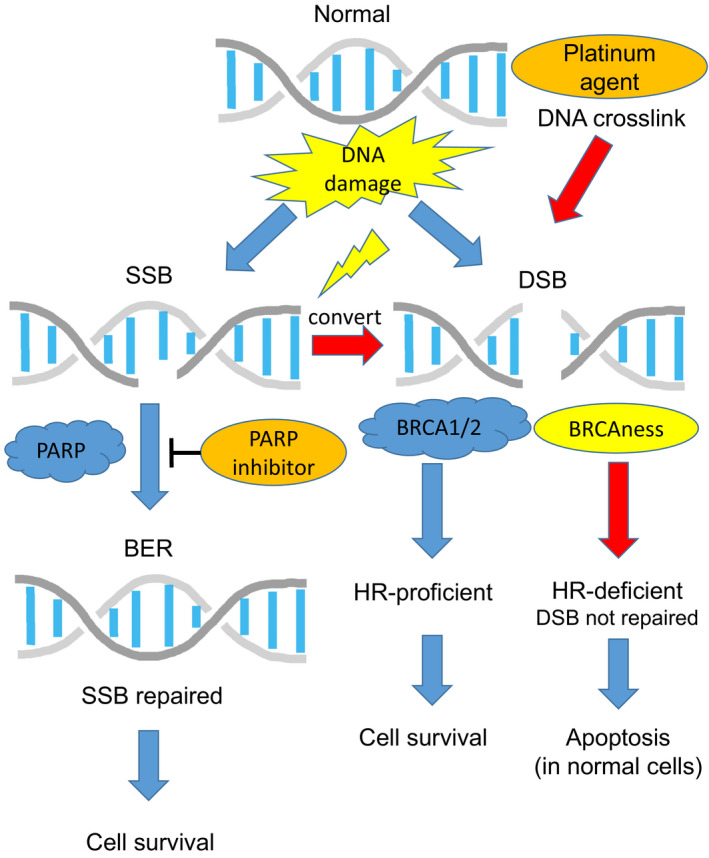
Overview of DNA damage repair pathways. A single‐strand break (SSB) is repaired by base excison repair (BER) via poly‐ADP ribose polymerase (PARP). PARP is a vital element of the BER pathway and plays a crucial role in sensing and binding to single‐strand DNA damage and results in the activation of catalytic proteins including topoisomerases, histones, and PARP itself for the repair of the DNA damage. For a cell that has a defective homologous recombination (HR) pathway such as BRCA1/2 mutations (BRCAness), the loss of ability to repair single‐strand DNA damage (PARP inhibition) could be lethal. Double‐strand breaks (DSBs) are fixed by HR via BRCA1/2. BRCA1/2‐deficient cells without HR ability store DBSs, resulting in genomic instability and an increased predisposition to play malignant behaviors. Platinum agents crosslink purine bases on DNA, thereby disturbing transcription and stopping replication, which lead to DSBs and the apoptosis. Cells with mutated HR genes (BRCAness) display hypersensitivity to crosslinking agents such as platinum agents

Platinum agents crosslink purine bases on DNA, thereby disturbing transcription and stopping replication, which lead to DSBs and apoptosis.^54^ Consequently, mutations in HR genes display hypersensitivity to DNA crosslinking agents (Figure 3). Indeed, PDAC cells with *BRCA2*, *FANCC*, or *FANCG* gene mutations exhibit hypersensitivity to DNA crosslinking agents such as cisplatin or mitomycin C in vitro and in vivo.^55^ PDAC cases with impaired DNA repair pathways revealed better response to platinum‐based chemotherapy and radiation therapy that induce DNA damage than those with normal DNA repair pathways.^16^, ^56^ It is noteworthy that structural variations in platinum agents, as has been observed for cisplatin and oxaliplatin, can be differences in DDR recognition. These differences in recognition influence the cytotoxicity of individual platinum agents.^57^

Sporadic PDAC patients with *BRCA1/2* mutation displayed worse survival after operation than those with wild‐type *BRCA*.^58^ On the other hand, platinum‐based chemotherapy notably improved survival outcome in patients with *BRCA1/2* mutations.^58^ Consequently, the survival differences relative to wild‐type patients were eliminated.^58^ In other studies, patients with *BRCA1/2* mutation displayed the enhanced response rates to platinum‐based chemotherapy and improved survival outcome.^16^, ^59^, ^60^, ^61^ In 25 stage IV PDAC patients, an additional cisplatin to the GnP regimen displayed complete responses in 8% and partial responses in 62.5%, with an OS of 16.5 months and 20% of patients alive at 2 years.^62^ Interestingly, most patients who responded to this treatment were enriched for *BRCA1* or *BRCA2* mutation‐related PDACs.^62^ Thus, DDR gene mutations confer hypersensitivity to platinum‐based chemotherapy.^16^ By the current uniform approach, there is a possibility that patients with DDR‐mutated tumors (up to 25%) are not optimally treated using platinum agents. Identification of these patients early in the course of disease leads to improving their survival outcomes. In addition, it is possible that patients with DDR‐mutated PDACs who have undergone resection would have a greater chance of being cured with an adjuvant platinum‐based regimen.

### 3.3 PARP inhibitors

SSBs are the most frequent DNA damage. If they are not repaired efficiently, they develop into DSBs.^44^, ^63^ The base excision repair (BER) pathway is an important repair machinery for SSBs (Figure 3). Poly‐ADP ribose polymerase (PARP) is a vital element of the BER pathway and plays a crucial role in sensing and binding to single‐strand DNA damage and results in the activation of catalytic proteins including topoisomerases, histones, and PARP itself for the repair of the DNA damage.^44^, ^64^, ^65^ For a cell lacking HR pathway such as *BRCA1/2* or *PALB2* mutations, disability to repair single‐strand DNA damage can be lethal. Preclinical study has showed evidence that PARP inhibitors (PARPi) abrogate DNA repair in HR‐defective cells including PDACs.^66^ *BRCA1*/*2* gene mutations have been reported to gift hypersensitivity to PARPi in preclinical models and early clinical trials of PDAC.^16^, ^67^ Clinical study has shown preliminary evidence that PARP inhibitors and platinum‐based agents have notable anti‐cancer effects in *BRCA*‐mutant PDACs.^51^ Lowery et al^68^ reported three partial responses and one prolonged stable disease among 15 *BRCA*‐mutated PDACs treated with PARPi‐based therapy. Kaufman et al^67^ reported one complete response and four partial responses among 23 *BRCA*‐mutated PDACs treated with olaparib (PARPi) monotherapy. Shroff et al^69^ reported a 16% response (one complete response and two partial responses) to rucaparib (PARPi) monotherapy in 19 *BRCA*‐mutated PDACs with more than 1 prior systemic treatment. Of interest, patients with somatic *BRCA* mutations also displayed a sensitivity to rucaparib (PARPi). A recent phase III trial of olaparib (the POLO trial) displayed considerable improvement in PFS in germline *BRCA*‐mutated metastatic PDAC patients who were sensitive to platinum agents in first‐line therapy.^70^ In addition, a recent study reported that *ATM*‐mutant PDAC cells were responsive to the olaparib (PARPi) or the ATR inhibitor VE‐822, and showed the that treatment with either of these inhibitors induced intense accumulation of DSBs and diminished tumor cell viability in vitro and in vivo.^71^ Thus, the subtype of PDAC patients with DDR deficiency is sensitive to platinum analogs and PARP inhibitors. Up to 40% of PDAC patients with germline DDR mutation do not have any significant family history to imply a predisposing disease such as breast or ovarian cancer.^72^ The present NCCN guidelines note that germline testing is recommended for all patients with PDAC.

### 3.4 DNA mismatch repair protein deficiency

Whole‐genome sequencing and whole‐exome sequencing of PDACs have revealed a mean mutation load of 1.8 and 1.1 mutation per megabase (Mb), respectively.^73^ Only 5% of PDACs exhibit the hypermutated phenotype.^73^ Rare tumors with >12 somatic mutations/Mb hold mismatch repair (MMR) deficiency, the cause of which has been associated with *MSH2* gene promoter deletion or mutation or *MLH1* gene promoter methylation.^73^ Tumors with a high tumor burden (4‐12 mutations/Mb) often hold HR repair deficiency.^73^ The identification of these hypermutated PDACs is important because patients with these tumors are applicable for immunotherapy.^73^ After testing 12 019 cancers, the prevalence of microsatellite instability was found to be around 5% in many solid tumors, while in PDAC it was only 2%.^74^ These MMR‐deficient tumors carried high neo‐antigen load and displayed considerably improved responses to programmed cell death 1 blockade.^74^ Immunotherapy is a rapidly progressing field in cancer treatment. Among the immunotherapy modalities, immune checkpoint inhibition has displayed considerable success in several solid tumors, but there is still no significant benefit in PDAC. Immune checkpoint inhibition has been shown to be hopeful in MMR‐deficient colorectal and other cancers.^75^, ^76^ Le et al^74^ showed that solid tumors with MMR deficiency are response to immune checkpoint blockade with pembrolizumab. Objective response by radiographic assessment was found in 53% of patients with MMR deficiency, and complete response was detectable in 21%.^74^ Pembrolizumab has subsequently been approved by the FDA for solid tumors with MMR deficiency, regardless of tissue of origin. Furthermore, the clinical benefit of pembrolizumab was confirmed in patients with microsatellite instability‐high/MMR‐deficient non‐colorectal cancers including pancreatic cancer.^77^ Even in patients with advanced non‐colorectal cancer who experienced failure with prior therapy, objective response rate to pembrolizumab and the median overall survival were 34.3% and 23.5 months, respectively.^77^

## 4 CONCLUSIONS

Advances in precision medicine for PDACs according to the genetics and molecular biology of this disease may represent the next alternative approach to overcome the heterogeneity of different patients and improve survival outcomes for this poor prognostic disease. Reclassifying tumors into subtypes according to the genetic and molecular profiles of PDAC may guide novel treatment decisions with biological and prognostic relevance. In particular, DNA repair dysfunction is a determinant of sensitivity to platinum agents and PARPi. The COMPASS study using real‐time WGS and RNA sequencing to identify molecular and genetic characterization of PDAC and facilitate better treatment selection for PDACs is ongoing.^20^ A deep understanding of the molecular and cellular crosstalk in the tumor microenvironment helps to establish scientifically rational treatment strategies for cancers that show specific molecular profiles. A combination of targeted therapies guided by molecular and genetic characterization of PDAC will be the ultimate therapeutic approach. Here we summarize the molecular subtypes or actionable gene mutations for precision medicine in PDAC patients, where we believe breakthroughs will soon emerge to fight this deadly disease.

## CONFLICTS OF INTEREST

The authors have no conflicts of interest to declare.

## ETHICAL STATEMENT

The authors are accountable for all aspects of the work in ensuring that questions related to the accuracy or integrity of any part of the work are appropriately investigated and resolved.
